# Abruptio Placenta among Pregnant Women Admitted to the Department of Obstetrics and Gynaecology in a Tertiary Care Centre: A Descriptive Cross-sectional Study

**DOI:** 10.31729/jnma.7796

**Published:** 2022-11-30

**Authors:** Sujata Maharjan, Meena Thapa, Babita Chaudhary, Subij Shakya

**Affiliations:** 1 Department of Obstetrics and Gynaecology, Kathmandu Medical College and Teaching Hospital, Sinamangal, Kathmandu, Nepal; 2 Department of Obstetrics and Gynaecology, Nepalese Army Institute of Health Sciences, Sanobharyang, Kathmandu, Nepal; 3 Kathmandu Medical College and Teaching Hospital, Sinamangal, Kathmandu, Nepal

**Keywords:** *abruptio placenta*, *epidemiology*, *fetal outcome*, *incidence*, *maternal outcome*

## Abstract

**Introduction:**

Abruptio placenta is the complete or partial separation of the normally implanted placenta before delivery of the foetus. It is one of the commonest causes of antepartum haemorrhage affecting maternal and foetal outcomes. Early detection and timely intervention of abruptio placenta in daily clinical practice are important to improve maternal and perinatal outcomes. The objective of the study was to find out the prevalence of abruptio placenta among the pregnant women admitted to the Department of Obstetrics and Gynaecology in a tertiary care centre.

**Methods:**

A descriptive cross-sectional study was done among the pregnant women admitted to the Department of Obstetrics and Gynaecology in a tertiary care centre where data from medical records was taken from 1 January 2021 to 31 December 2021 after taking ethical approval from the Institutional Review Committee (Reference number: 1102202208). Demographic details of the patients including age and parity were recorded. Convenience sampling was done. Point estimate and 95% Confidence Interval were calculated.

**Results:**

Out of 1514 deliveries, abruptio placenta was seen in 10 (0.66%) (0.25-1.07, 95% Confidence Interval) cases.

**Conclusions:**

The prevalence of abruptio placenta among pregnancies was similar to the studies done in similar settings.

## INTRODUCTION

Abruptio placenta is classically defined as the complete or partial separation of a normally implanted placenta before delivery of the fetus.^1^ Placental separation occurs due to rupture of a uterine spiral artery leading to bleeding into decidua basalis expanding as retroplacental hematoma causing compromise of the blood supply to the fetus.^2^ Abruption occurs in 0.4-1% of pregnancies. Obstetric haemorrhage accounts for 1/3^rd^ of maternal death. Perinatal mortality is high with abruption due to its strong association with preterm.^1^

Abruptio placenta is one of the catastrophic obstetric conditions with adverse maternal and perinatal outcomes. Early detection and timely intervention can be done in our daily clinical practice.

This study aimed to find out the prevalence of abruptio placenta among the pregnant women admitted to the Department of Obstetrics and Gynaecology in a tertiary care centre.

## METHODS

This descriptive cross-sectional study was conducted from medical records among the pregnant women admitted to the Department of Obstetrics and Gynaecology of Kathmandu Medical College and Teaching Hospital, Sinamangal, Kathmandu from 1 January 2021 to 31 December 2021. Ethical approval was obtained from the Institutional Review Committee (Reference number: 1102202208). Convenience sampling was used. The sample size was calculated using the following formula:


$$\begin{array}{ll} \text{n} & ={\text{Z}}^{2}\times \frac{\text{p}\times \text{q}}{{\text{e}}^{\text{2}}}\\ & =1.96^{2}\times \frac{0.50\times 0.50}{0.03^{2}}\\ & =1068 \end{array}$$

Where,

n= minimum required sample size
Z= 1.96 at 95% Confidence Interval (CI)
p= prevalence taken as 50% for maximum sample size calculation
q= 1-p
e= margin of error, 3%

The minimum sample size calculated was 1068. By adding a 10% non-response rate, the total sample size was 1187. However, final sample size taken was 1514.

A proforma was created following which data were collected retrospectively from hospital records Inclusion criteria includes all pregnancies diagnosed as abruptio placenta. Exclusion criteria were pregnancies with vaginal bleeding, abdominal pain and decreased fetal movement causes other than abruptio placenta. Demographic details of the patients including age, and parity were recorded. Documented history, general physical examination and obstetric examination were reviewed. Clinical presentation of patients like vaginal bleeding, pain abdomen, and decreased foetal movements were collected along with maternal highrisk factors like pregnancy-induced hypertension (PIH), gestational diabetes mellitus (GDM), polyhydramnios, prelabour rupture of membrane (PROM), trauma, smoking was noted. Total deliveries during the aforementioned period were taken into consideration to calculate the prevalence of abruptio placenta. Classically, abruptio placenta is diagnosed clinically when pregnant women have a sudden onset of abdominal pain, vaginal bleeding, and uterine tenderness. Unfortunately, laboratory tests or other diagnostic methods like sonography have limited use.^3^ In our context diagnosis was done clinically followed by retroplacental clots and couvelaire uterus during caesarean section.

Data were analysed using Microsoft Excel 2016 and IBM SPSS Statistics version 18.0. Point estimate and 95% CI were calculated.

## RESULTS

Out of 1514 deliveries, abruptio placenta was seen in 10 (0.66%) (0.25-1.07, 95% Confidence Interval) cases. The mean age of patients was 30±3.5 years. In most cases, 9 (90%) were between the ages of 20-34 years. A total of 8 (80%) of patients were multipara, while 2 (20%) were primipara. In terms of gestational age at delivery, 5 (50%) had a preterm delivery, and the other 5 (50%) delivered at term (Table 1).

**Table 1:** Demographic characteristics of cases (n = 10).

Characteristics	n (%)
**Maternal age (in years)**	
<20	-
20-34	9 (90)
≥35	1 (10)
**Parity**	
Primipara	2 (20)
Multipara	8 (80)
**Gestational age at delivery(weeks)**	
28-37	5 (50)
≥37	5 (50)

Among 10 patients, 4 (40%) presented with pain in the abdomen, 1 (10%) with Pervaginal (PV) bleeding, 2 (20%) with both pain abdomen and PV bleeding, and 2 (20%) with decreased foetal movement (Table 2).

**Table 2:** Clinical presentation and underlying condition among the pregnancies with abruptio placenta (n= 10).

Clinical presentation	n (%)
Pain abdomen	4 (40)
PV bleeding	1 (10)
Pain abdomen and PV bleeding	2 (20)
Decreased foetal movement	2 (20)
**Underlying conditions**	
PIH	3 (30)
PIH and GDM	2 (20)
Smoking	1 (10)
PROM	1 (10)
None	3 (30)

Maternal complications occurred in 4 (40%) of cases, evenly distributed among haemorrhagic shock 2 (20%) and Couvelaire uterus 2 (20%) (Table 3).

**Table 3:** Maternal complications and mode of delivery (n= 10).

Characteristics	n (%)
**Maternal complications**	
Haemorrhagic shock	2 (20)
Couvelaire uterus	2 (20)
**Mode of delivery**	
Vaginal delivery	5 (50)
Caesarean delivery with indications	5 (50)
• Foetal heart rate abnormalities	2 (20)
• Retroplacental blood clot by Ultrasound	2 (20)
• Bloody amniotic fluid	1 (10)

In terms of foetal outcome, 1 (10%) foetus was admitted to neonatal intensive care unit (NICU) who was discharged at 28 days of life (Table 4).

**Table 4:** Foetal outcome.

Viability	n (%)
Stillbirth	5 (50)
Live birth	5 (50)
NICU admission	1 (10)

## DISCUSSION

Abruptio placenta is abnormal bleeding from small uterine arteries into decidua basalis.^4^ The most probable factor causing abruptio placenta is an insufficient trophoblastic invasion.^5^ It is one of the serious complications of pregnancy affecting both maternal and foetal outcomes.

The prevalence of abruptio placenta in this study was 0.66% which is similar to the study done in the United States.^6^ Similar studies within the Indian subcontinent, Tanzania reported the incidence of abruptio placenta as 3-5 per 1000 deliveries.^7-10^

Clinical presentation of abruptio placenta depends upon the severity of bleeding and degree of separation of placenta after 28 weeks of gestation. Most studies report vaginal bleeding as a common presentation of abruptio placenta.^11-13^ In contrast to our study where the common presentation was pain abdomen (40%) followed by pain abdomen and vaginal bleeding (20%).

One of the most important risk factors for placental abruption is definitely the hypertensive disorders of pregnancy and has been supported by previous studies reported.^9,14,15^ However, smoking was an important risk factor identified in 45% of the women in another study done in Pakistan.^12^ This variation may be due to the geographical and socioeconomic differences in the study sites. In our study 30% had pregnancy-induced hypertension, 20% had PROM, 10% were smokers, 20% had PIH with GDM and 20% had no underlying condition.

Among our patients, 50% had vaginal deliveries and 50% had caesarean deliveries. Similar findings were reported in the study done in Pakistan while in India and Finland, a high percentage of caesarean delivery was observed at 90.32% and 91 % respectively.^8,12,13^ Difference in the mode of delivery is due to the viability of the foetus at the time of presentation. In our study, 50% of cases presented as intrauterine foetal death (IUFD) at the time of presentation who underwent induction for vaginal delivery.

Placental abruption is associated with increased maternal complications. Development of disseminated intravascular coagulation (DIC) along with an increase in transfusion requirement due to severe blood loss and consequent development of consumption coagulopathy were found in abruptio placenta. Moreover, in placental abruption cases, the frequencies of hysterectomy, maternal death, pulmonary oedema, and renal failure were also reported.^16^ In a study conducted in Thailand in 2006, among 103 cases most common complication of abruptio placenta was a hemorrhagic shock (19.4%) followed by Couvelaire uterus (16.5% ) and DIC (5.8%).^2^ We observed in our study that 20% of cases had a hemorrhagic shock and 20% had a Couvelaire uterus. Life-threatening maternal complications like DIC, renal failure, shock, pulmonary oedema, and infections were not documented. The lower rate of complication could be due to timely intervention at the time of presentation.

Placental abruption is associated with preterm labour, low birth weight, and foetal demise. The perinatal mortality and morbidity of abruptio placenta depend on the gestational week at which abruption developed.^17^ Regarding foetal outcome, we observed that 50% of babies were born alive while 50% were stillbirth. Among 50% of stillbirths, 80% were preterm and 60% had low birth weight supporting foetal outcome depending upon the period of gestation. The percentage of preterm deliveries is consistent with that of other studies.^13,17^

This study has some limitations. The number of recorded cases was small. Conduction of related study for longer duration with greater size would have represented the target population.

## CONCLUSIONS

The prevalence of abruptio placenta among pregnancies is similar to the studies done in similar settings. Abruptio placenta is one of the most common causes of antepartum haemorrhage affecting maternal and foetal outcomes. Abruptio placenta should be suspected whenever the patient presents with pain in the abdomen with/without PV bleeding, decreased foetal movement along with detection of bloody amniotic fluid, and foetal heart rate abnormalities. The timely intervention of abruptio placenta reduces maternal and perinatal mortality.
